# Correlates of walking and cycling for transport and recreation: factor structure, reliability and behavioural associations of the perceptions of the environment in the neighbourhood scale (PENS)

**DOI:** 10.1186/1479-5868-10-87

**Published:** 2013-07-02

**Authors:** Emma J Adams, Anna Goodman, Shannon Sahlqvist, Fiona C Bull, David Ogilvie

**Affiliations:** 1 British Heart Foundation National Centre for Physical Activity and Health, School of Sport, Exercise and Health Sciences, Loughborough University, Loughborough, UK; 2 London School of Hygiene and Tropical Medicine, London, UK; 3 Centre for Physical Activity and Nutrition Research (C-PAN), School of Exercise and Nutrition Sciences, Deakin University, Geelong, Australia; 4 Centre for the Built Environment and Health, School of Population Health, The University of Western Australia, Perth, Australia; 5 Medical Research Council Epidemiology Unit and UKCRC Centre for Diet and Activity Research (CEDAR), Institute of Public Health, University of Cambridge, Cambridge, UK

**Keywords:** Walking, Cycling, Transport, Recreation, Urban environment, Measurement, Reliability

## Abstract

**Background:**

Emerging evidence suggests that walking and cycling for different purposes such as transport or recreation may be associated with different attributes of the physical environment. Few studies to date have examined these behaviour-specific associations, particularly in the UK. This paper reports on the development, factor structure and test-retest reliability of a new scale assessing perceptions of the environment in the neighbourhood (PENS) and the associations between perceptions of the environment and walking and cycling for transport and recreation.

**Methods:**

A new 13-item scale was developed for assessing adults’ perceptions of the environment in the neighbourhood (PENS). Three sets of analyses were conducted using data from two sources. Exploratory and confirmatory factor analyses were used to identify a set of summary environmental variables using data from the iConnect baseline survey (n = 3494); test-retest reliability of the individual and summary environmental items was established using data collected in a separate reliability study (n = 166); and multivariable logistic regression was used to determine the associations of the environmental variables with walking for transport, walking for recreation, cycling for transport and cycling for recreation, using iConnect baseline survey data (n = 2937).

**Results:**

Four summary environmental variables (traffic safety, supportive infrastructure, availability of local amenities and social order), one individual environmental item (street connectivity) and a variable encapsulating general environment quality were identified for use in further analyses. Intraclass correlations of these environmental variables ranged from 0.44 to 0.77 and were comparable to those seen in other similar scales. After adjustment for demographic and other environmental factors, walking for transport was associated with supportive infrastructure, availability of local amenities and general environment quality; walking for recreation was associated with supportive infrastructure; and cycling for transport was associated only with street connectivity. There was limited evidence of any associations between environmental attributes and cycling for recreation.

**Conclusion:**

PENS is acceptable as a short instrument for assessing perceptions of the urban environment. Previous findings that different attributes of the environment may be associated with different behaviours are confirmed. Policy action to create supportive environments may require a combination of environmental improvements to promote walking and cycling for different purposes.

## Background

Although the health benefits of participating in regular physical activity have been well established [1], only 39% of men and 29% of women in England meet recommended guidelines [2]. The physical environment has been identified in ecological models as an important influence on physical activity behaviour [3]. Therefore identifying attributes of the physical environment associated with higher levels of physical activity could help provide pointers for changing the environment to support a sustainable increase in physical activity participation. A number of such attributes have been shown to be associated with participation in physical activity in general, or with specific types of activity such as walking and cycling in local neighbourhoods [4-7].

Emerging evidence suggests that different attributes of the environment are associated with activities being undertaken for different purposes, such as walking and cycling for transport or for recreation [8-12]. For example, reviews suggest that walking for transport is associated with density, land use mix, street connectivity and proximity of destinations [10,12], while walking for recreation or leisure is associated with pedestrian infrastructure, land use mix, personal safety and aesthetics [10]. Fewer studies have explored environmental attributes and their associations with cycling for transport or recreation. The presence of dedicated cycle routes or paths, separation of cycling from other traffic, high population density and proximity of a cycle path or green space have been found to be positively associated with cycling for transport [13]. This behaviour has also been reported to be positively related to a ‘cyclability’ index including proximity to destinations, good walking and cycling facilities, difficulty in parking near shopping areas and aesthetics [14]. Evidence from European studies suggests positive relationships between cycling for transport and residential density, land use mix, street connectivity, access to destinations and degree of urbanisation, and some evidence for a relationship between cycling for recreation and walking and cycling infrastructure [12].

Understanding the relationships between different characteristics of the physical environment and participation in specific behaviours is important for informing transport and planning policy and practice with the regards to the development of ‘activity friendly’ environments that support different types of activity. However, to date, few studies have examined physical environment attributes and their association with walking and cycling behaviours separately and for different purposes. Many studies have assessed only one of the behaviours (e.g. walking or cycling), one behaviour for one specific purpose (e.g. walking for transport), or one all-encompassing behavioural outcome (e.g. total walking or total cycling). In addition, most studies have been conducted in the USA and Australia, where the context may differ from that of other countries in terms of urban design, land use and transport infrastructure. Studies that assess relationships of different attributes of the physical environment with each of the four behaviours separately are therefore needed, particularly in countries such as the UK.

When conducting physical activity research it is often necessary to collect detailed data on a variety of complex behaviours (physical activity, travel, sedentary time) as well as on proposed individual, social and environmental influences on those behaviours. However, excessively long questionnaires can discourage participation in research [15,16] and it is therefore important to develop instruments that assess the factors of interest using as few items as possible. This paper reports on the development of a new, short scale to assess perceptions of the environment in the neighbourhood (PENS), including its factor structure and test-retest reliability, and its application to examine which characteristics of the neighbourhood physical environment are associated with the likelihood of participating in walking and cycling for transport and recreation as separate behaviours in the UK. This study was undertaken as part of the iConnect project, a five-year natural experimental study that aims to assess the impact of improving walking and cycling infrastructure on travel, physical activity and carbon emissions [17].

## Methods

The study involved four stages: (1) development of a new scale to assess the perceptions of the environment in the neighbourhood (PENS); (2) factor analysis of PENS using data from the iConnect baseline survey to determine which items measured similar constructs and thereby create a reduced set of summary environmental variables; (3) test-retest analysis to assess the reliability of the individual items and environmental factors in PENS using data collected through a separate reliability study; and (4) an examination of the associations between perceptions of the environment and four specific behaviours: walking for transport, walking for recreation, cycling for transport and cycling for recreation using data from the iConnect baseline survey.

### Survey data

#### ***Core iConnect baseline survey: participants and procedures***

Baseline data for the core module of the iConnect study were collected in April 2010 using a postal survey in three UK study areas: Cardiff, Kenilworth and Southampton. The evaluation framework, survey methods and survey contents have been reported elsewhere [17,18]. In brief, 22,500 adults living in the study areas were randomly selected to take part from the edited electoral register for those areas. Participants were mailed a survey pack containing a letter of invitation, a copy of the survey, a consent form and a freepost return envelope. Participants who did not respond within two weeks were sent a second survey pack as a reminder. Comparisons with local and national data indicated that the 3516 participants who completed the survey (16% response rate) tended to be older than the local population and had a somewhat higher level of educational attainment and better health. Otherwise they appeared to be broadly representative in their demographic, socio-economic and travel characteristics [19].

#### ***Reliability study: participants and procedures***

Participants were recruited for a separate study to assess the reliability of the survey items. In October 2010, 3000 adults were randomly selected from the edited electoral register for six wards in the town of Loughborough, UK and invited to complete the iConnect survey on two separate occasions, approximately seven days apart. The initial mailing contained a letter of invitation, a copy of the survey, a consent form and a freepost return envelope. Individuals who completed and returned the first survey (n = 216) were then immediately posted the second survey. Participants who did not return the second survey within seven days received reminder phone calls or letters. A total of 166 individuals completed surveys 1 and 2. A prize draw to win one of twenty £25 gift vouchers was offered as an incentive for participation for those who completed both surveys.

Both the core survey and the reliability study received ethical approval from the University of Southampton Ethics Committee (CEE 200809-15).

### Measures

#### ***Perceptions of the neighbourhood environment***

Perceptions of the ‘neighbourhood environment’ (specified in the questionnaire to mean the area within a 10-15 minute walk from the respondent’s home) were measured using 13 items selected and adapted from the 49-item ALPHA (Assessing Levels of Physical Activity and Fitness) European environmental questionnaire [20]. Items were selected through a process of identifying the factors purporting to be the most relevant environmental influences for walking or cycling for transport or recreation and included: safety from traffic (three items); safety from crime (one item); street connectivity (three items); infrastructure (two items); destinations (two items); and aesthetics (two items) (Table 1). Respondents were asked to indicate their level of agreement on a five point Likert scale from ‘strongly agree’ to ‘strongly disagree’. Responses to the negatively worded items (8/13) were subsequently reverse coded and relabelled such that a high score represented a perception of a highly supportive environment, and responses to all items were then coded on a scale of -2 (low support) to +2 (high support) and given ‘short item names’ for convenience in reporting (Table 1).

**Table 1:** Survey items assessing environmental perceptions of the neighbourhood, short item name and question source

**Q1. Firstly, we’d like to ask you about the neighbourhood where you live. By neighbourhood we mean the area that you could walk to in 10-15 minutes from your home. How much do you agree with the following statements about your neighbourhood?**			
**Survey item assessing perceptions of the neighbourhood environment**^**a**^	**Short item name**^**c**^	**Measure**	**Source: adapted from ALPHA**^**d**^
a) Walking is unsafe because of the traffic	Walking safe from traffic	Safety from traffic	Q5c
b) Cycling is unsafe because of the traffic	Cycling safe from traffic	Safety from traffic	Q5d
c) There are no convenient routes for walking and cycling	Convenient walk/cycle routes	Street connectivity	Q7a/b
d) There are not enough safe places to cross roads	Safe to cross roads	Safety from traffic	Q5b
e) The area is unsafe because of the level of crime or anti-social behaviour	Area safe from crime	Safety from crime	Q5e/f
f) The area is generally free from litter or graffiti^b^	Free from litter	Aesthetics	Q6b (response scale amended)
g) There are places to walk or cycle to, for example: shops, restaurants, leisure facilities^b^	Places to walk/cycle to	Destinations	Q2a-g: collapsed to one item/response scale amended
h) There are open spaces, for example: parks, sports fields or beaches^b^	Open spaces	Destinations	Q2h
i) There are pavements suitable for walking^b^	Pavements for walking	Infrastructure	Q3a
j) There are special lanes, routes or paths for cycling^b^	Cycle lanes/routes	Infrastructure	Q3c
k) There are many road junctions^b^	Many road junctions	Street connectivity	Q7c
l) There are many different routes for walking and cycling so I don’t have to go the same way every time^b^	Variety of walk/cycle routes	Street connectivity	Q7d
m) The area is pleasant for walking or cycling^b^	Pleasant to walk/cycle	Aesthetics	Q6a

^a^Assessed using a five point Likert scale from strongly agree to strongly disagree; ^b^Items were reverse coded for use in analyses; ^c^Items were coded such that a high score = a highly supportive environment; ^d^ALPHA = European environmental questionnaire [20].

#### ***Walking and cycling for transport and recreation***

We examined associations between environmental perceptions and past-week participation in four behaviours: walking for transport, walking for recreation, cycling for transport and cycling for recreation. Past-week participation in walking and cycling for transport was measured as part of a set of questions relating to travel behaviour, which was assessed across five categories of trip purpose: to and from work; for business purposes; to and from a place of study; for shopping and personal business; and to visit friends or family or for other social activities. For each journey purpose participants were asked to report the total time spent (in hours and minutes) and the total distance travelled in the last seven days using each of six different modes of transport (walking, cycling, bus, train, car (as a driver) and car (as a passenger) plus ‘other’, which captured modes such as taxi and van). Individuals who reported walking or cycling for any journey purpose were treated as having participated in walking or cycling for transport. Walking and cycling for recreation were assessed using items adapted from the International Physical Activity Questionnaire (IPAQ) [21], which asked participants to report any participation in these behaviours over the past week.

#### ***Individual characteristics***

Sex, age, ethnic group, educational qualifications, housing tenure, and number of cars and bicycles in the household were reported in the survey. Residential location (urban or rural) for participants in the core baseline survey was assigned to each participant using home postcode and the 2004 Rural and Urban Area Classification to match Lower Super Output Areas to settlement type [22].

### ***Analyses***

Three sets of analyses were conducted to assess: (1) the factor structure of the environmental perceptions items in PENS; (2) the test-retest reliability of the individual environmental perceptions items in PENS and the environmental factors identified in the factor analysis; and (3) the associations of the environmental factors with the likelihood of participating in each of the four target behaviours.

#### ***Factor structure of environmental perception items***

In the core baseline sample, the factor structure of the environmental perception items was explored to determine which items measured similar constructs, and thereby create a reduced set of summary environmental variables. Data from the core baseline survey were randomly split into two halves. In the first ‘derivation’ half, exploratory factor analyses (EFAs) were conducted in MPlus5 (Muthén & Muthén, Los Angeles), specifying stratification by iConnect site (Cardiff, Southampton or Kenilworth). In these EFAs the MPlus extension for ordinal data was used [23], rotating the factor loadings using an oblique geomin rotation in order to allow the latent factors to correlate [24]. The factor structures indicated by the EFA were then tested by fitting confirmatory factor analyses (CFAs) in the second ‘test’ half of the core baseline survey sample. Multi-group CFA analyses were fitted to verify that any selected model structure showed measurement invariance (i.e. the same relationship between manifest and latent variables) across the iConnect sites [25]. These CFAs were conducted using multivariate probit analysis with the extension for ordinal data and estimating model fit using the Weighted Least Squares, mean and variance adjusted (WLSMV) estimator. In both the EFA and CFA analyses, pairwise present estimation was used to include individuals with missing data for some items (n = 331).

**Figure 1:**
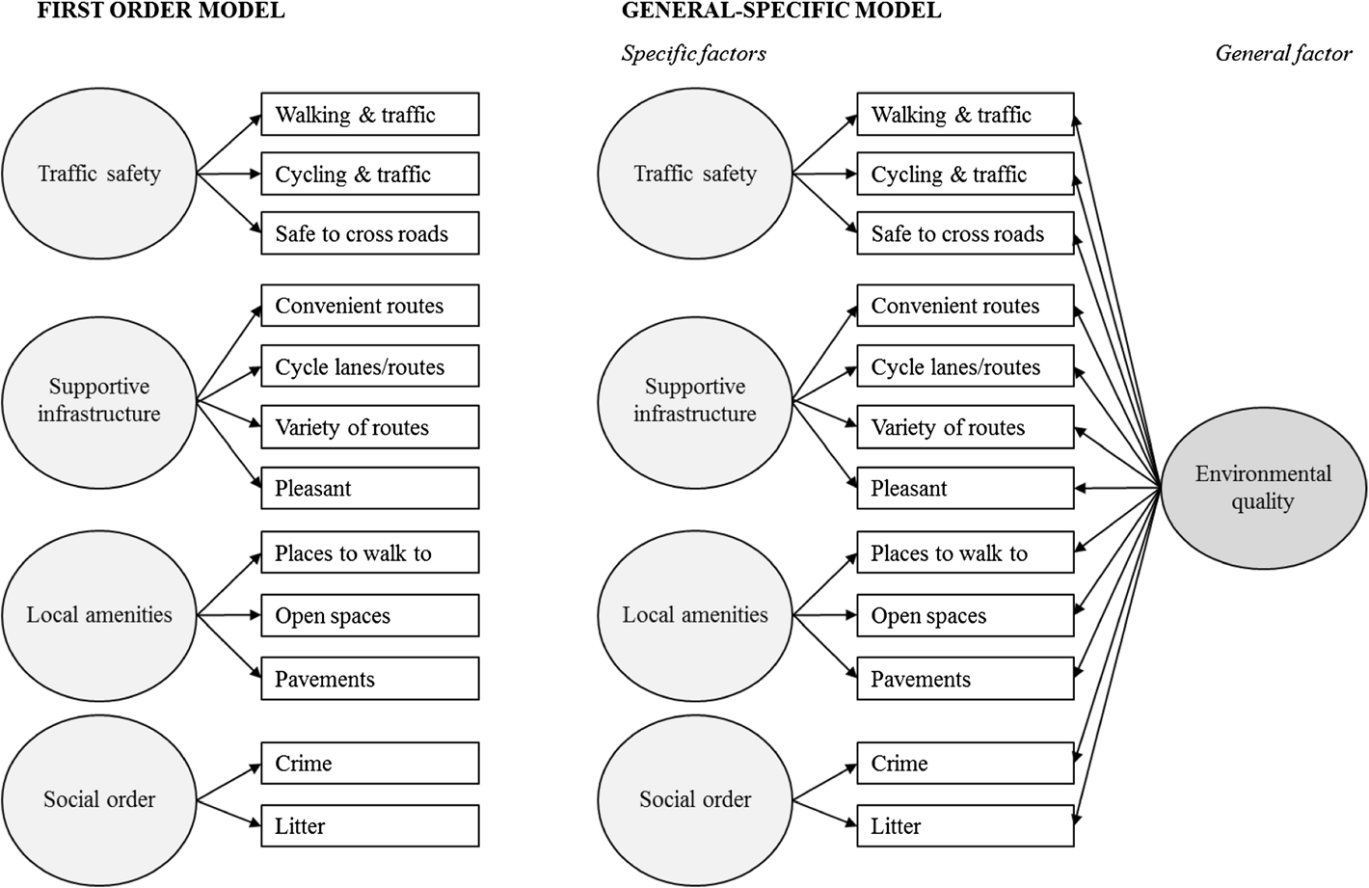
First order and general-specific models fitted in multi-group confirmatory factor analyses of environmental perception items.

The fit of first-order and general-specific models (see examples of each in Figure 1) were compared, thereby examining whether adding a general ‘environment quality’ scale (which included all 13 items from PENS in one summary scale) improved model fit over and above taking account of specific subscales. Common practice was followed in reporting multiple indices of fit, namely the Comparative Fit Index (CFI), the Tucker Lewis Index (TLI) and the Root Mean Square Error of Approximation (RMSEA) [26,27]. We considered a model with CFI > 0.90, TLI > 0.90 and RMSEA < 0.08 to have acceptable fit, and a model with CFI > 0.95, TLI > 0.95 and RMSEA < 0.05 to have good fit [26]. The standardised loadings of each variable onto its factor(s) were checked to ensure they were of reasonable magnitude (>0.4). Where models showed acceptable fit on some indices but not on others, correlations between the unique variances of some individual items were permitted [28].

The above analyses were conducted using the original, five-point set of item responses. As a sensitivity analysis, the analyses were repeated after collapsing the responses into a three-point scale of strongly disagree/disagree; neutral; and agree/strongly agree. The results were very similar and any minor differences are noted in the text.

#### ***Test-retest reliability of the environmental perceptions itemsM***

In the reliability study sample, intraclass correlation coefficients (ICC) for the thirteen individual items in PENS (on five-point scales) and the mean scores of each of the environmental factors identified in the factor analysis were used to compute the coefficient of stability of the scores on the two tests. An ICC ≥0.75 was considered as good reliability, ICC 0.5 to <0.75 represented moderate reliability and an ICC of <0.5 represented poor reliability [29]. Data were analysed using SPSS Statistics (version 19.0) (IBM SPSS Inc, Armonk, New York).

#### ***Associations between environmental factors and walking and cycling for transport and recreation***

In the core baseline sample, mean scores were calculated for all environmental factors identified in the factor analysis by taking the average across all constituent variables with non-missing data. These mean scores were then recoded into three categories of environmental supportiveness with cut points equally spread across the range of scores: low support (- 2 to -0.67); medium support (-0.66 to +0.66); and high support (+0.67 to +2). Logistic regression was used to examine the associations between each environmental factor and spending any time in the past week walking for transport, walking for recreation, cycling for transport or cycling for recreation (measured as four separate outcomes). Two models were fitted: Model 1 which included adjustment for sex (male, female), age (<30, 30-44, 45-64, ≥65 years), ethnic group (white, other), education (degree, ‘A’ Level or equivalent, GCSE grades A to C, or less), housing tenure (owned, rented from private landlord, rented from local authority, other), household cars (0, 1, 2 or more), household bicycles (0, 1 or more), residential location (urban or rural), and case study site (Cardiff, Kenilworth, Southampton) and Model 2 which added further adjustment for each of the other environmental factors in the analysis (low, medium, high support). Respondents (n = 2937) were only included if data were available for all environmental factors, all four behaviours and all covariates included in the statistical models. Data were analysed in SPSS Statistics (version 19.0) (IBM SPSS Inc, Armonk, New York).

## Results

### Participant characteristics

Table 2 shows the demographic characteristics of participants included in the three sets of analyses. The characteristics of those included in the factor analysis (n = 3494) and the analysis of the associations between environmental factors and walking and cycling for transport and recreation (n = 2937) were similar to those of the total sample for the baseline survey (n = 3516) (data not shown).

**Table 2:** Sample characteristics

	**iConnect baseline survey**				**Reliability study**	
**Characteristic**	**Respondents included in factor analysis**		**Respondents included in environmental correlates analyses**			
	**n = 3494**		**n = 2937**		**n = 166**	
	**%**	**n**^**a**^	**%**	**n**	**%**	**n**^**a**^
**Sex**						
Male	45.1	1560	45.6	1338	46.7	77
**Age (years)**						
<30	16.3	554	18.0	530	11.7	19
30-44	20.5	699	22.3	656	24.5	40
45-64	38.9	1324	39.5	1161	36.2	59
≥65	24.3	827	20.1	590	27.6	45
**Ethnicity**						
White	94.7	3220	95.2	2796	90.7	147
Other	5.3	179	4.8	141	9.3	15
**Education**						
Degree	40.7	1371	42.9	1259	31.7	51
GCE ‘A’ Level	17.8	599	18.9	556	17.4	28
GCSE Grades A to C	18.7	631	18.4	540	24.2	39
No formal qualification	22.8	766	19.8	582	26.7	43
**Housing tenure**						
Owned	74.7	2555	75.5	2218	81.1	133
Rented from private landlord	14.9	508	15.9	468	6.1	10
Rented from local authority	7.6	260	6.0	177	11.6	19
Other	2.8	97	2.5	74	1.2	2
**Household cars**						
0	13.4	457	12.2	359	12.3	20
1	41.1	1406	39.5	1159	43.8	71
2 or more	45.6	1559	48.3	1419	43.8	71
**Household bicycles**						
0	42.3	1382	40.9	1201	34.3	57
1 more	57.7	1888	59.1	1736	65.7	109
**Residential location**						
Urban	95.4	3333	95.7	2812	n/a	n/a
Rural	4.6	161	4.3	125	n/a	n/a
**Site**						
Cardiff	32.0	1118	31.8	934	n/a	n/a
Kenilworth	32.0	1119	32.5	955	n/a	n/a
Southampton	36.0	1257	35.9	1048	n/a	n/a

^a^Numbers do not sum up to a total due to missing responses; n/a: not applicable.

### Factor analysis

#### ***Exploratory factor analysis***

In the sample of 3494 individuals, the item ‘many road junctions’ showed the lowest correlation with other items (mean Pearson correlation -0.01, range -0.15 to 0.12), while the items ‘convenient walk/cycle routes’ and ‘pleasant to walk/cycle’ showed the highest correlation (mean 0.29 for each, range 0.02 to 0.54). Full details of the inter-item correlations are presented in Additional file 1: Table A1).

In the ‘derivation’ half of the sample, exploratory factor analysis (EFA) indicated that there were four factors with an Eigenvalue of 1 or above (4.3, 2.0 and 1.3 and 1.0), and therefore both three- and four-factor solutions were examined. The three-factor solutions showed somewhat poorer model fit than the four-factor solutions in the EFA, including non-acceptable fit on some model indices. The three-factor solution also made less sense conceptually and appeared to be tapping into valences, with factor 2 capturing ‘bad things about the area’ and factor 3 capturing ‘good things about the area’(see Additional file 2: Table A2). The four-factor solution had greater face validity in terms of bringing together items relating to similar aspects of the environment across all three sites (data not shown).

One complication in choosing the four-factor solution was that the two items which showed the highest mean correlation with other items also consistently loaded onto two factors. ‘Pleasant to walk/cycle’ loaded on both factor 2 and factor 3, but loaded more strongly on factor 3. This was thought to make more sense conceptually because of its specific reference to walking and cycling; this item was therefore assigned to factor 3. ‘Convenient walk/cycle routes’ loaded more strongly on factor 1 than factor 4 when using the five-point Likert scale classification and loaded more strongly on factor 4 than factor 1 in the collapsed, three-point sensitivity analysis. This item made more sense conceptually on factor 4, and it was suspected that the factor 1 loading partly reflected position and valence artefacts (i.e. the fact that these questions were all asked together and all negatively worded); this item was therefore assigned to factor 4, but included in the model residual correlation between the unique variance of this item and those on factor 1. Finally, the item ‘many road junctions’ never loaded >0.4 which, in conjunction with its low correlation with other items, led to it being omitted from the environmental factors.

#### ***Confirmatory factor analysis***

Based on the EFA analyses, confirmatory factor analysis (CFA) was used on the ‘test’ half of the sample to evaluate the factor structures shown in Figure 1 and presented in detail in Table 3. All model indices showed acceptable fit in multi-group analyses (CFI = 0.938, TFL = 0.966, RMSEA = 0.078 in the first-order model; CFI = 0.967, TFL = 0.976, RMSEA = 0.065 in the general-specific model), suggesting measurement invariance across study sites. Results were similar in sensitivity analyses using the collapsed three-point scale, and measurement invariance was also shown in multi-group analyses by gender (CFI = 0.945, TFL = 0.969, RMSEA = 0.074 in the first-order model; CFI = 0.968, TFL = 0.976, RMSEA = 0.066 in the general-specific model). In the pooled sample, all standardised item loadings were acceptable or near-acceptable (all ≥0.38 on first-order factors; all ≥0.43 on either specific or general factor in general-specific model: see Table 3). Correlation of the subscales in the first-order model were moderate (0.46-0.58) for all subscale pairs except for routes and amenities, and even here the correlation of 0.75 did not suggest prohibitively high collinearity. Taken together, these results therefore led to the conclusion that the proposed four-factor structure showed good fit to the data, and this provided the basis for further analyses.

**Table 3:** **Fully standardised item loadings from CFA analyses of environmental perception items models**^**a**^

		**First-order (pooled sample**^**b**^**)**	**General-specific (pooled sample**^**b**^**)**	
			**Specific factors**	**General factor**
**STANDARDISED LOADINGS**				
**Factors**	**Items**^**†**^			
Traffic safety	Walking safe from traffic	0.81	0.67	0.50
	Cycling safe from traffic	0.70	0.59	0.43
	Safe to cross roads	0.71	0.47	0.48
Supportive infrastructure	Convenient walk/cycle routes	0.58	0.29	0.52
	Cycle routes	0.48	0.52	0.39
	Variety of walk/cycle routes	0.69	0.25	0.64
	Pleasant to walk/cycle	0.82	0.10	0.79
Local amenities	Places to walk/cycle to	0.74	0.52	0.57
	Open spaces	0.71	0.55	0.54
	Pavements for walking	0.76	0.32	0.63
Social order	Area safe from crime	0.79	0.31	0.49
	Free from litter	0.38	0.58	0.25
**CORRELATION OF SUBSCALES**		Traf with Soc: 0.53	[all correlations automatically set at zero in general-specific models]	
		Traf with Infr: 0.58		
		Traf with Am: 0.48		
		Soc with Infr: 0.52		
		Soc with Am: 0.46		
		Infr with Am: 0.75		

^a^Fitting models shown in Figure 1; ^b^n = 1754, ‘test’ half of the core baseline sample; Traf = Traffic safety score, Soc = Social order score, Infr = Supportive infrastructure score, Am = Local amenities score.

### Test-retest reliability of PENS

The mean score and test-retest reliability for the 13 individual items in PENS and the environmental factors identified in the factor analysis are shown in Table 4. The intraclass correlation (ICC) of individual items ranged from 0.32 (places to walk/cycle to) to 0.71 (area safe from crime). Seven items scored between 0.5 and 0.75, indicating moderate reliability (walking safe from traffic, cycling safe from traffic, safe to cross roads, convenient walk/cycle routes, presence of cycle lanes/routes, pleasant to walk/cycle, area safe from crime). Six items scored less than 0.5, indicating poor reliability (variety of walk/cycle routes, places to walk/cycle to, open spaces, pavements for walking, free from litter and many road junctions), although only two of these (places to walk/cycle to and pavements for walking) had an ICC of less than 0.45.

**Table 4:** **Test-retest reliability of mean scores for individual items assessing environmental perceptions and environmental factors**^**a**^

**Item name**^**b**^	**T1/T2**	**n**	**Mean (SD)**	**ICC**	**Environmental factor**	**T1/T2**	**n**	**Mean score (SD)**	**ICC**
Walking safe from traffic	T1	165	0.90 (1.13)	0.69	**TRAFFIC SAFETY**	T1	165	0.54 (1.04)	0.77
	T2		0.79 (1.08)			T2		0.47 (0.99)	
Cycling safe from traffic	T1	163	0.23 (1.32)	0.67					
	T2		0.13 (1.21)						
Safe to cross roads	T1	165	0.49 (1.23)	0.68					
	T2		0.51 (1.14)						
Convenient walk/cycle routes	T1	159	1.01 (1.14)	0.55	**SUPPORTIVE INFRASTRUCTURE**	T1	165	0.75 (0.78)	0.63
	T2		0.89 (0.96)			T2		0.71 (0.73)	
Cycle lanes/routes	T1	164	0.74 (1.15)	0.53					
	T2		0.76 (1.06)						
Variety of walk/cycle routes	T1	164	0.41 (1.09)	0.45					
	T2		0.50 (0.97)						
Pleasant to walk/cycle	T1	164	0.85 (1.01)	0.61					
	T2		0.71 (1.01)						
Places to walk/cycle to	T1	163	0.87 (1.14)	0.32	**LOCAL AMENITIES**	T1	165	1.07 (0.85)	0.42
	T2		0.83 (1.02)			T2		1.09 (0.79)	
Open spaces	T1	161	1.06 (1.12)	0.48					
	T2		1.12 (0.93)						
Pavements for walking	T1	162	1.30 (0.96)	0.36					
	T2		1.33 (0.86)						
Area safe from crime	T1	163	0.85 (1.13)	0.71	**SOCIAL ORDER**	T1	165	0.57 (0.93)	0.64
	T2		0.82 (1.06)			T2		0.62 (0.84)	
Free from litter	T1	162	0.30 (1.27)	0.47					
	T2		0.41 (1.09)						
Many road junctions	T1	162	0.72 (0.97)	0.45	**STREET CONNECTIVITY**	T1	162	0.72 (0.97)	0.45
	T2		0.65 (0.78)			T2		0.65 (0.78)	
All items (except ‘many road junctions’)					**ENVIRONMENT QUALITY**	T1	165	0.75 (0.61)	0.75
						T2		0.73 (0.61)	

^a^Reliability study sample (n = 166); ^**b**^Items were coded such that a high score = perceptions of a highly supportive environment; T1 = survey time point 1, T2 = survey time point 2, SD = standard deviation; ICC = Intraclass correlation coefficient; all ICCs presented p < 0.001 for difference from zero.

ICC for the environmental factors ranged from 0.42 (local amenities) to 0.77 (traffic safety). Two of the environmental factors (traffic safety and environment quality) had an ICC of >0.75 indicating good reliability. Two factors (supportive infrastructure and social order) had an ICC between 0.5 and 0.75 indicating moderate reliability and one environmental factor (local amenities) had an ICC less than 0.5 indicating poor reliability.

### Environmental associations with walking and cycling for transport and recreation

**Figure 2:**
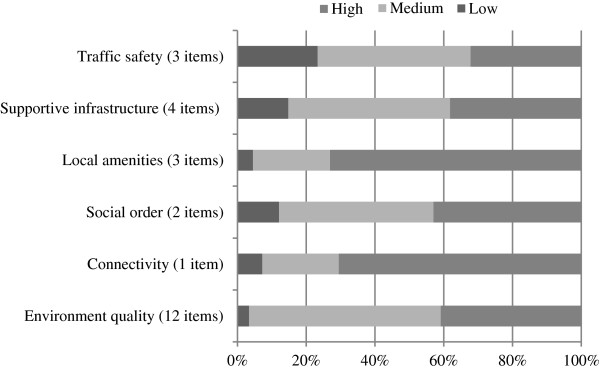
**Distribution of levels of support for environmental factors**^**a**^**. **^a^ Includes participants in environmental correlates analysis (n = 2937). Items = number of individual survey items included in environmental factor.

The distribution of low, medium and high support for each of the environmental factors in the core iConnect baseline sample is shown in Figure 2. The proportion of respondents reporting low support for the environmental factors ranged from 3.4% (environment quality) to 23.3% (traffic safety); medium support from 22.3% (street connectivity) to 55.7% (environment quality), and high support from 32.2% (traffic safety) to 73.1% (local amenities).

### Walking

Participation in walking for either purpose was similar, with 66.0% of respondents reporting any walking for transport (n = 1939) and 59.7% reporting any walking for recreation (n = 1753). The associations between perceptions of the environment and the likelihood of participating in any walking for transport or recreation are shown in Table 5.

**Table 5:** Environmental factors and likelihood of participating in any walking for transport or recreation

			**WALKING FOR TRANSPORT**				**WALKING FOR RECREATION**			
**n = 2937**^**a**^			**MODEL 1**^**b**^		**MODEL 2**^**c**^		**MODEL 1**^**b**^		**MODEL 2**^**c**^	
			**OR (CI)**	**p-value**	**OR (CI)**	**p-value**	**OR (CI)**	**p-value**	**OR (CI)**	**p-value**
**Traffic safety**										
(3 items)	Low		1.0		1.0		1.0		1.0	
	Medium		1.19 (0.97-1.47)	0.091	1.07 (0.86-1.32)	0.561	1.05 (0.86-1.27)	0.647	0.97 (0.79-1.19)	0.765
	High		1.16 (0.93-1.45)	0.185	0.98 (0.77-1.26)	0.902	1.02 (0.83-1.25)	0.883	0.82 (0.65-1.03)	0.089
**Supportive infrastructure**										
(4 items)	Low		1.0		1.0		1.0		1.0	
	Medium		1.52 (1.20-1.91)	0.001^#^	1.35 (1.05-1.73)	0.018*	0.99 (0.79-1.23)	0.913	1.0 (0.788-1.26)	0.972
	High		1.65 (1.30-2.10)	0.000^#^	1.44 (1.08-1.90)	0.012*	1.41 (1.12-1.77)	0.004^#^	1.47 (1.13-1.92)	0.005^#^
**Local amenities**										
(3 items)	Low		1.0		1.0		1.0		1.0	
	Medium		1.85 (1.24-2.75)	0.003^#^	1.71 (1.14-2.58)	0.010^#^	0.94 (0.64-1.38)	0.741	0.94 (0.63-1.38)	0.736
	High		2.46 (1.68-3.58)	0.000^#^	2.14 (1.44-3.18)	0.000^#^	1.16 (0.81-1.67)	0.417	1.06 (0.73-1.56)	0.754
**Social order**										
(2 items)	Low		1.0		1.0		1.0		1.0	
	Medium		0.81 (0.61-1.08)	0.146	0.77 (0.58-1.02)	0.072	1.15 (0.90-1.47)	0.254	1.12 (0.88-1.43)	0.366
	High		0.86 (0.65-1.15)	0.321	0.77 (0.57-1.03)	0.081	1.12 (0.87-1.43)	0.391	1.06 (0.82-1.37)	0.675
**Street connectivity**										
(1 item)	Low		1.0		1.0		1.0		1.0	
	Medium		1.05 (0.75-1.46)	0.791	0.95 (0.67-1.330	0.750	0.94 (0.68-1.30)	0.698	0.89 (0.64-1.23)	0.480
	High		1.18 (0.87-1.61)	0.277	1.05 (0.76-1.43)	0.782	1.03 (0.77-1.39)	0.836	0.96 (0.71-1.30)	0.799
**General environment quality**										
(12 items)	Low		1.0		-	-	1.0		-	-
	Medium		1.91 (1.24-2.94)	0.003^#^	**-**	-	0.85 (0.56-1.30)	0.449	-	-
	High		2.32 (1.49-3.60)	0.000^#^	**-**	**-**	1.08 (0.71-1.66)	0.716	-	-

^a^iConnect core baseline sample; ^b^adjusted for sex, age, ethnic group, education, housing tenure, household cars, household bicycles, location (urban or rural) and case study site; ^c^adjusted for b and the environmental factors which are not the independent variable in the analysis; *p < 0.05 #p < 0.01; OR = odds ratio; CI = 95% confidence interval.

#### ***Walking for transport***

Participants who reported medium or highly supportive infrastructure were significantly more likely to report walking for transport than those who reported low support. This association attenuated slightly in the second model but remained significant (medium support: OR (odds ratio) 1.35, 95% CI 1.05 to 1.73; high support: OR 1.44, 95% CI 1.08 to 1.90). A similar pattern was observed for the availability of local amenities with those reporting high availability being around twice as likely to walk for transport than those reporting low availability (medium support: OR 1.71, 95% CI 1.14 to 2.58; high support: OR 2.14, 95% CI 1.44 to 3.18). In our analyses of a combined ‘general environment quality’ summary scale, participants who perceived the general environment to be of medium or high quality were significantly more likely to walk for transport compared to those who perceived the environment quality to be low (medium support: OR 1.91, 95% CI 1.24 to 2.94; high support: OR 2.32, 95% CI 1.49 to 3.60).

#### ***Walking for recreation***

A positive significant association was observed between supportive infrastructure and the likelihood of doing any walking for recreation in both models, but only for participants who perceived the infrastructure to be highly supportive (model 2: OR 1.47, 95% CI 1.13 to 1.92). Associations with the other environmental factors were non-significant.

### Cycling

Participation in cycling for either purpose was low, with 13.1% reporting any cycling for transport (n = 386) and 13.5% reporting any cycling for recreation (n = 397). The associations between perceptions of the environment and the likelihood of participating in any cycling for transport and recreation are shown in Table 6.

**Table 6:** Environmental factors and likelihood of participating in any cycling for transport or recreation

		**CYCLING FOR TRANSPORT**				**CYCLING FOR RECREATION**			
**n = 2937**^**a**^		**MODEL 1**^**b**^		**MODEL 2**^**c**^		**MODEL 1**^**b**^		**MODEL 2**^**c**^	
		**OR (CI)**	**p-value**	**OR (CI)**	**p-value**	**OR (CI)**	**p-value**	**OR (CI)**	**p-value**
**Traffic safety**									
(3 items)	Low	1.0		1.0		1.0		1.0	
	Medium	0.91 (0.66-1.24)	0.530	0.83 (0.60-1.16)	0.274	0.81 (0.60-1.10)	0.178	0.82 (0.60-1.12)	0.205
	High	1.01 (0.73-1.40)	0.952	0.89 (0.61-1.29)	0.530	0.97 (0.71-1.33)	0.851	0.91 (0.64-1.31)	0.620
**Supportive infrastructure**									
(4 items)	Low	1.0		1.0		1.0		1.0	
	Medium	1.04 (0.73-1.48)	0.821	0.98 (0.67-1.42)	0.902	0.73 (0.53-1.02)	0.065	0.68 (0.48-0.97)	0.031*
	High	1.19 (0.83-1.71)	0.341	1.09 (0.72-1.66)	0.693	0.96 (0.68-1.34)	0.799	0.82 (0.55-1.22)	0.326
**Local amenities**									
(3 items)	Low	1.0		1.0		1.0		1.0	
	Medium	1.56 (0.75-3.26)	0.232	1.51 (0.72-3.18)	0.280	0.90 (0.47-1.71)	0.736	0.98 (0.51-1.89)	0.955
	High	1.83 (0.91-3.70)	0.090	1.69 (0.82-3.49)	0.156	1.25 (0.68-2.23)	0.473	1.38 (0.73-2.58)	0.321
**Social order**									
(2 items)	Low	1.0		1.0		1.0		1.0	
	Medium	1.05 (0.70-1.57)	0.811	1.05 (0.70-1.57)	0.808	1.28 (0.84-1.94)	0.251	1.30 (0.85-1.98)	0.228
	High	1.33 (0.88-2.01)	0.181	1.32 (0.86-2.01)	0.202	1.44 (0.94-2.20)	0.098	1.44 (0.93-2.22)	0.104
**Street connectivity**									
(1 items)	Low	1.0		1.0		1.0		1.0	
	Medium	1.95 (1.04-3.65)	0.038*	1.93 (1.02-3.63)	0.042*	0.75 (0.45-1.25)	0.269	0.77 (0.46-1.29)	0.323
	High	1.69 (0.93-3.06)	0.082	1.64 (0.90-2.99)	0.106	0.97 (0.61-1.54)	0.899	0.97 (0.60-1.55)	0.887
**General environment quality**									
(12 items)	Low	1.0		-	-	1.0		-	-
	Medium	0.78 (0.40-1.50)	0.450	-	-	0.54 (0.30-0.99)	0.046*	**-**	-
	High	0.98 (0.50-1.91)	0.956	-	-	0.61 (0.33-1.13)	0.116	-	-

^a^iConnect core baseline sample; ^b^adjusted for sex, age, ethnic group, education, housing tenure, household cars, household bicycles, location (urban or rural) and case study site; ^c^adjusted for b and the environmental factors which are not the independent variable in the analysis; *p < 0.05 #p < 0.01; OR = odds ratio; CI = 95% confidence interval.

#### ***Cycling for transport***

In both models, participants who perceived there to be medium street connectivity had twice the odds of cycling for transport than those who perceived there to be low street connectivity (model 1: OR 1.95, 95% CI 1.04-3.65; model 2: OR 1.92, 95% CI 1.02 to 3.63). Perceptions of medium and high availability of local amenities were associated with a non-significant increased likelihood of cycling for transport. Associations with the other environmental factors were also non-significant.

#### ***Cycling for recreation***

Participants were more likely to cycle for recreation if they perceived social order to be medium or high rather than low, however this association was non-significant. Findings for the other environmental factors were mixed with significant negative associations being observed for a medium level of supportive infrastructure (model 2) and overall general environment quality (model 1). Associations with the other environmental factors were non-significant.

## Discussion

A new short scale for assessing adults’ perceptions of the environment in the neighbourhood (PENS) was developed to enable the attributes of the neighbourhood environment which are associated with the likelihood of participating in walking and cycling for transport and recreation as separate behaviours to be examined for the UK.

### Perceptions of the environment in the neighbourhood scale (PENS)

Although a number of instruments have previously been developed for assessing perceptions of the neighbourhood environment [30,31], many of them are long and the wording of items is not necessarily applicable to all countries and settings. Survey length has been found to be important in influencing response rate [15,16] and thus shorter instruments are needed, particularly when a large number of constructs are being measured, to reduce overall survey length. In order to reduce participant burden and tailor items to the UK neighbourhood environment, the 49-item ALPHA instrument [20] was modified and a new much shorter 13-item instrument, the perceptions of the environment in the neighbourhood scale (PENS), was developed for use in the iConnect project.

The factor structure of the items in PENS was explored to identify items measuring similar constructs and create a set of summary variables. Four environmental factors (traffic safety, supportive infrastructure, local amenities and social order) showed a good fit to the data and these were used for further analyses along with a single item, street connectivity. The factors were found to have acceptable test-retest reliability (ICC range 0.42 to 0.77). A general environment quality scale (which included 12 of the 13 items in the scale) was also computed. Its reliability was found to be good (ICC = 0.75) and similar to that found in other studies, e.g. that of a summary neighbourhood score assessing perceived environmental characteristics associated with active travel (ICC = 0.73) [32].

Reliability of individual items assessed in the scale ranged from ICC 0.32 to 0.71. These findings are similar to those of studies of other questionnaires, in which ICC ranged from 0.54 to 0.76 [20] and 0.33 to 0.70 [32]. Comparing items with similar wording and response scales in both questionnaires, seven items in PENS with moderate reliability (ICC >0.5) also had moderate reliability in the ALPHA study [20]. Of the others, two items in PENS had slightly poorer reliability than in the ALPHA questionnaire despite similar wording (‘variety of walk/cycle routes’ and ‘many road junctions’). Three of the PENS items (‘free from litter’, ‘places to walk/cycle to’ and ‘pavements for walking’) performed less well than the related ALPHA questions. This could be explained by differences in the response scale for ‘free from litter’ (ALPHA: none, a few, some, plenty; PENS: five points from strongly agree to strongly disagree); by subtle differences in wording for ‘pavements for walking’ (PENS: suitable pavements for walking; ALPHA: any pavements); and by differences in both response scale and wording for ‘places to walk/cycle.’ Specifically, in PENS one single item was used to assess access to destinations whereas in ALPHA respondents were asked about how long it would take to walk to each of seven different destinations. Overall, PENS was found to have acceptable reliability with similar variation in reliability across individual items and composite measures to other surveys assessing perceptions of the neighbourhood environment [33-37]. PENS may therefore offer an acceptable, shorter alternative for measuring the perceptions of the neighbourhood environment using only a few items.

### Environmental perceptions and likelihood of participation in walking and cycling for transport or recreation

Our results support emerging findings from recent research which suggest that different attributes of the physical environment are associated with participation in walking and cycling being undertaken for different purposes [8-12]. In our study, walking for transport was positively associated with supportive infrastructure, availability of local amenities and general environment quality, whereas walking for recreation was positively associated only with supportive infrastructure. Cycling for transport was associated only with street connectivity, and cycling for recreation was not significantly associated with any of the environmental attributes we assessed.

#### ***Walking***

Our finding that supportive infrastructure was positively related to walking for transport is consistent with findings from a recent review [11]. Availability of local amenities or destinations has been reported to be positively related to walking for transport in a number of reviews [5,10,11,38,39] and our study supports these findings. The lack of any relationship between perceived traffic safety and walking for transport seen in our study has also been reported elsewhere [5,7,10,11] although another review has reported a positive relationship in studies adjusting for neighbourhood self-selection [40].

Our finding that few environmental attributes were associated with walking for recreation has also been reported in a number of other studies [10-12]. One possible explanation is that recreational walking may more typically extend in distance or be done outside of the neighbourhood, which may explain why stronger associations with environmental attributes have more often been demonstrated with transport-related physical activity than with recreational physical activity [5,6,12,38].

#### ***Cycling***

Fewer studies have investigated the environmental correlates of cycling than those of walking. In our study street connectivity was the only environmental exposure found to be significantly positively associated with cycling for transport. Street connectivity relates to the ease of travel between two points and is directly related to street design. It is important for cycling for transport because it determines: route directness (which affects journey distance and therefore duration); route choice (whether it is possible to take different routes to get to and from the same destination); and reflects among other things, the potential presence of barriers such as motorways or cul-de–sacs which can affect both directness and choice [6]. Road network designs providing high connectivity have been found to be associated with greater cycling for transport in two other studies [41,42]. Our other findings for cycling for transport are consistent with those of a recent review which found no relationships with traffic safety, social order (crime safety) and aesthetics [12]. However, in contrast to our study, others have found cycling for transport to be related to access to local amenities [12] and supportive infrastructure (cycling facilities, dedicated cycle paths or routes) [12,13,41,43].

Cycling for recreation was not strongly associated with any of the environmental attributes in our study, although there was tentative evidence of possible relationships with supportive infrastructure (negative), social order (positive) and general environment quality (negative). These results are largely consistent with the findings from a recent review of European studies which found limited associations between recreational cycling and environmental attributes except for traffic-related safety [12].

Our limited findings in relation to cycling may reflect the relatively small proportion of participants in our study (less than 14%) who reported any cycling. In addition, given that it is possible to travel much further and beyond the neighbourhood when cycling, environmental attributes in the immediate surroundings of the home may be less important in influencing whether a person cycles or not than environmental factors assessed at a larger scale, such as the characteristics of commuting routes and destinations. Other individual, social and cultural factors, which may be stronger predictors of participation in cycling than environmental factors, also need to be considered [41,44,45]. The need to explore the relationships between environmental attributes and different behaviours at different geographic scales, as well the relationships between the individual, social, environmental and cultural factors, remain an important area for future investigation.

### Limitations

We relied on self-report measures for assessing perceptions of the physical environment and participation in walking and cycling which may have resulted in both error and bias in their measurement. Objective measurement of physical activity behaviours and attributes of the physical environment can be used to overcome some of these measurement issues, however challenges remain with the collection and analysis of these types of data [30] and it was not feasible to use them within this large cohort study.

Although separate models were used to estimate associations for each specific behaviour of interest, the environmental attributes were not assessed separately for walking and cycling. Composite measures were used to assess environmental constructs, which may have reduced the effect of individual environmental attributes. In some cases the items included in summary variables seemed unintuitive, for example the item ‘pavements for walking’ was included in the local amenities factor rather than the supportive infrastructure factor. The use of these composite measures also made it difficult to compare our findings to those of other studies for some attributes. In addition, whereas our measures of environmental perceptions were restricted to the neighbourhood, our measures of the behaviours asked about all walking and cycling for transport and recreation (not restricted to the neighbourhood). We may therefore have underestimated the associations between the environmental attributes and the behaviours of interest, as evidence suggests that the predictive capacity of models appears to improve with increased specificity for environmental and behavioural measures and the setting in which the behaviour takes place [46].

Other potential sources of bias in this study include the relatively low response rates and the possibility of neighbourhood self-selection, whereby individuals who are more active choose to live in neighbourhoods that support their preferences. We were not able to measure or adjust for neighbourhood self-selection in this study because of constraints on the length of our questionnaire.

The survey was conducted in only three case study sites which may differ from other areas, towns and cities and therefore limit the generalizability of our findings. Limited environmental heterogeneity, particularly in terms of street connectivity and safety, may have limited power to detect associations with the behaviours. Future studies might consider sampling from areas with greater heterogeneity. Finally, due to the cross sectional nature of this study, the causal relationships between perceptions of the environment and walking and cycling behaviours cannot be inferred. Longitudinal studies are needed to help determine any causal relationships.

## Conclusions

This study is one of few studies to have examined attributes of the physical environment and their associations with walking and cycling behaviours separately and for different purposes. A new, short (13-item) scale (PENS) was developed for assessing perceptions of the environment in the neighbourhood. Individual items and summary variables derived from PENS were found to have acceptable test-retest reliability and PENS may therefore provide researchers with a suitable short instrument for use in future studies. The results from this study confirm that, as has been observed in other countries, different attributes of the environment in the UK may be important for supporting participation in different physical activity behaviours being undertaken for different purposes. The practical implication is that creating supportive ‘activity friendly’ environments is likely to require a combination of environmental improvements to promote walking and cycling for different purposes. Although single-fix solutions might appear superficially attractive, our findings reinforce those of a growing body of research that suggest that one size does not fit all. Improvements to walking and cycling infrastructure, access to destinations and therefore land use mix, as well as the general environmental quality should all be taken into account in future transport and planning policy.

## Competing interests

The authors declare that they have no competing interests.

## Authors’ contributions

All authors contributed to the design of different parts of the studies. EA, FB, DO and SS were involved in the development of the iConnect core survey. FB and EA designed the reliability study and led the data collection. EA analysed the reliability study data. EA, AG, DO and FB were involved in the analyses of the environmental correlates data. AG conducted the factor analysis and drafted the associated sections of this manuscript. EA drafted the remainder of the manuscript and all other authors critically reviewed and revised versions of the manuscript. All authors read and approved the final manuscript.

## Supplementary Material

Additional file 1: Table A1 Pearson correlations between environmental perception items and mean values. Click here for file

Additional file 2: Table A2 Results of exploratory factor analysis for environmental perception items. Click here for file
