# Supplementary material for: Correlates of walking and cycling for transport and recreation: factor structure, reliability and behavioural associations of the perceptions of the environment in the neighbourhood scale (PENS)
Source: Int J Behav Nutr Phys Act. 2013 Jul 2;10:87. doi: 10.1186/1479-5868-10-87 (PMC3702387; doi:10.1186/1479-5868-10-87)
Supplement: Additional file 1: Table A1 — Pearson correlations between environmental perception items and mean values. [file 1479-5868-10-87-S1.pdf]

ADDITIONAL FILE 1

Table A1: Pearson correlations between environmental perception items and mean values[a]

		A	B	C	D	E	F	G	H	I	J	K	L	M	Mean absolute correlation
A	Walking safe from traffic	1													0.26
B	Cycling safe from traffic	0.53	1												0.26
C	Convenient walk/cycle routes	0.47	0.54	1											0.29
D	Safe to cross roads	0.52	0.42	0.52	1										0.25
E	Area safe from crime	0.34	0.25	0.25	0.28	1									0.19
F	Free from litter	0.08	0.08	0.04	0.06	0.27	1								0.12
G	Places to walk/cycle to	0.18	0.14	0.22	0.17	0.09	0.15	1							0.08
H	Open spaces	0.13	0.04	0.14	0.12	0.14	0.14	0.47	1						0.19
I	Pavements for walking	0.27	0.16	0.24	0.21	0.15	0.15	0.40	0.39	1					0.22
J	Cycle lanes/routes	0.10	0.22	0.31	0.18	0.02	0.03	0.22	0.16	0.18	1				0.16
K	Many road junctions	-0.09	-0.15	-0.07	-0.10	-0.11	-0.08	0.12	0.12	0.11	0.09	1			0.01
L	Variety of walk/cycle routes	0.20	0.24	0.32	0.22	0.12	0.09	0.31	0.28	0.27	0.31	0.10	1		0.23
M	Pleasant to walk/cycle	0.27	0.31	0.35	0.26	0.32	0.24	0.31	0.38	0.32	0.28	-0.02	0.48	1	0.29

[a]n=3494, missing data 1.7-2.5% for all items
