# Supplementary material for: Correlates of walking and cycling for transport and recreation: factor structure, reliability and behavioural associations of the perceptions of the environment in the neighbourhood scale (PENS)
Source: Int J Behav Nutr Phys Act. 2013 Jul 2;10:87. doi: 10.1186/1479-5868-10-87 (PMC3702387; doi:10.1186/1479-5868-10-87)
Supplement: Additional file 2: Table A2 — Results of exploratory factor analysis for environmental perception items. [file 1479-5868-10-87-S2.pdf]

ADDITIONAL FILE 2

Table A2. Results of exploratory factor analysis for environmental perception items[a]

Original (5-point) item categories	Three factor[b,f]			Four factor[c,f]			
	**Factor 1**	**Factor 2**	**Factor3**	**Factor 1**	**Factor 2**	**Factor3**	**Factor 4**
Walking safe from traffic		**-0.82**					**0.78**
Cycling safe from traffic		**-0.76**				0.34	**0.67**
Safe to cross roads		**-0.71**					**0.65**
Convenient walk/cycle routes	-0.30	**-0.63**				**0.43**	**0.61**
Cycle routes	-0.39		0.32			**0.52**	
Variety of walk/cycle routes			**0.57**			**0.60**	
Pleasant to walk/cycle			**0.53**		**0.47**	**0.64**	
Places to walk/cycle to			**0.72**	**-0.67**			
Open spaces			**0.79**	**-0.75**			
Pavements for walking			**0.61**	**-0.66**			
Area safe from crime	**0.48**	**-0.58**			**0.55**		0.33
Free from litter	0.36				**0.52**		
Many road junctions			0.35	-0.36			

Collapsed (3-point) item categories	Three factor[d,f]			Four factor[e,f]			
	**Factor 1**	**Factor 2**	**Factor3**	**Factor 1**	**Factor 2**	**Factor3**	**Factor 4**
Walking safe from traffic		**0.79**		0.44			**0.95**
Cycling safe from traffic		**0.80**				**0.41**	**0.64**
Safe to cross roads		**0.68**					**0.60**
Convenient walk/cycle routes		**0.75**				**0.65**	**0.53**
Cycle routes	-0.37		**-0.43**			**0.60**	
Variety of walk/cycle routes			**-0.53**			**0.55**	
Pleasant to walk/cycle			**-0.60**		**0.50**	**0.54**	
Places to walk/cycle to			**-0.71**	**0.62**			
Open spaces			**-0.81**	**0.74**			
Pavements for walking			**-0.61**	**0.70**			
Area safe from crime	**0.56**	0.35			**0.56**		0.32
Free from litter	**0.44**				**0.56**		
Many road junctions							

[a] 'Derivation' half of the core baseline sample used for analyses (n=1740); [b] CFI= 0.905, TLI=0.924 RMSEA=0.102; [c] CFI=0.975, TLI=0.976, RMSEA=0.058; [d] CFI= 0.936, TLI=0.933 RMSEA=0.061; [e] CFI=0.983, TLI=0.978, RMSEA=0.035; [f] Loadings over 0.3 reported, loadings over 0.4 presented in bold.
